# Twelve‐Year Follow‐Up of a Randomised Controlled Trial Comparing the Effectiveness of Pelvic Floor Muscle Training Versus Mid‐Urethral Sling Surgery for Female Moderate to Severe Urinary Incontinence

**DOI:** 10.1111/1471-0528.18092

**Published:** 2025-02-11

**Authors:** Hélène F. C. van Oorschot, Deodata Tijsseling, Julien Labrie, Carl H. van der Vaart

**Affiliations:** ^1^ Department of Women and Baby University Medical Centre Utrecht (UMCU) Utrecht the Netherlands; ^2^ Department of Gynecology Bergman Clinics Hilversum the Netherlands; ^3^ Department of Gynecology Spaarne Gasthuis Haarlem the Netherlands

**Keywords:** follow‐up, improvement, midurethral sling surgery, pelvic floor muscle therapy, physiotherapy, PORTRET, randomised controlled trial, surgery, tension free vaginal tape, urinary incontinence

## Abstract

**Objective:**

To compare the 12‐year effectiveness of pelvic floor muscle training versus midurethral sling surgery for moderate to severe female stress urinary incontinence.

**Design:**

Observational follow‐up study of a randomised controlled trial.

**Setting:**

Conducted at the Division of Gynaecology, University Medical Centre Utrecht, The Netherlands.

**Population:**

Women from the PORTRET study experiencing moderate to severe stress urinary incontinence.

**Methods:**

A validated questionnaire was sent to participants.

**Main Outcome Measures:**

The primary outcome was subjective improvement in urinary incontinence symptoms. Secondary outcomes included subjective cure, severity of incontinence, impact of incontinence as urogenital symptom and cross‐over and re‐operation rates.

**Results:**

In this long‐term study, 184 of 386 (47.7%) women responded to the questionnaire. Cross‐over (86.9%) from the initial physiotherapy group to surgery was very high. No statistically significant differences were found in the intention to treat analysis. However, the post hoc analysis showed that women who underwent physiotherapy only reported a statistically significant lower improvement compared to those who underwent initial surgery (50.6% absolute difference; 95% CI 28.2–73.1) or surgery after physiotherapy (49.7% absolute difference; 95% CI 25.8–73.7). Subjective cure, decrease in perceived severity and impact of urinary incontinence also statistically significantly favoured women who underwent (initial) surgery Re‐operation was reported by 4.6% of women.

**Conclusion:**

This 12‐year follow‐up study showed a very high cross‐over rate to surgical treatment, considering a substantial proportion of non‐responders. Midurethral sling surgery, either initial or after physiotherapy, statistically significantly improved subjective outcomes for moderate to severe stress urinary incontinence as compared to pelvic floor muscle physiotherapy only in the long‐term.

## 1 Introduction

Stress urinary incontinence (SUI) is a common condition that has a negative impact on the quality of life for millions of women worldwide [1, 2]. It is defined by the International Continence Society as: ‘The complaint of any involuntary loss of urine on effort or physical exertion (e.g sporting activities) or on sneezing or coughing’ [3]. Pelvic floor muscle training (physiotherapy) and midurethral‐sling surgery (surgery) are both primary treatment modalities for SUI.

In the Physiotherapy OR Tvt Randomised Efficacy Trial (PORTRET), the clinical‐ and cost‐effectiveness of physiotherapy versus surgery as primary treatment for moderate to severe SUI were compared. At 12 months follow‐up, outcomes of the PORTRET study favoured women who received surgery, either initially or after physiotherapy, compared to physiotherapy only. Furthermore, after 12 months the frequency of cross‐over to surgery was 49.0% and a re‐operation was performed in 7/314 (2.2%) of women [4].

Studies focusing on the long‐term effectiveness of sling surgery report high subjective cure rates [5, 6, 7]. Regarding long term effectiveness of physiotherapy, literature is scarce and outcomes are heterogeneous [8, 9]. Long‐term follow‐up of midurethral sling surgery and pelvic floor muscle therapy compared in a randomised controlled trial has not been performed to date. The aim of our study is to present long‐term outcomes of the PORTRET study.

## 2 Methods

### 2.1 Study Design

We performed a long‐term follow‐up of the PORTRET study using patient reported outcome measures. Questionnaires were sent by post to patients who were previously enrolled in the original study. For this long‐term follow‐up, women from participating centres with at least four inclusions in the PORTRET study were included. Protocol of the PORTRET study design, in‐ and exclusion criteria, the randomisation process and surgical and physiotherapy techniques have been published previously [4, 10].

Initial physiotherapy and initial surgery were compared in an intention to treat analysis. Since at the initial 12 months follow‐up already 49.0% of women had crossed over from physiotherapy to surgery, we did not only focus on the intention to treat analysis, but also on a post hoc analysis [4]. In this post hoc analysis three treatment modalities were compared; women who underwent surgery, women who underwent surgery after physiotherapy and women who underwent physiotherapy only.

### 2.2 Outcomes

The primary outcome was defined as subjective improvement of urinary incontinence, measured with the use of the validated Patient Global Impression of Improvement (PGI‐I) [11, 12]. This questionnaire uses a 7‐point Likert scale that ranks the response to a single question from ‘very much worse’ to ‘very much better.’ In concordance with other studies, improvement was considered to be clinically significant if the patient's response was ‘much better’ or ‘very much better’, thus responses were dichotomised into ‘improvement’ and ‘no improvement’ [13, 14, 15].

Secondary outcomes included severity of incontinence, subjective cure, and urogenital symptoms. The Patient Global Impression of Severity (PGI‐S) was used to assess severity of incontinence on a 4‐point Likert scale. Responses were dichotomised into ‘no symptoms’ and ‘symptoms’ (mild, moderate, or severe) [13]. Subjective cure was defined as a negative response to the question from the validated Dutch version of the Urogenital Distress Inventory (UDI): ‘Do you experience urine leakage related to physical activity, coughing, or sneezing?’ [16, 17] Responses were dichotomised into ‘cure and “no cure”’. Lastly, urogenital symptoms were also measured with the validated Dutch version of the UDI [16, 17] The five UDI domains analysed were: urinary incontinence, overactive bladder, obstructive micturition, discomfort or pain and genital prolapse. Change from baseline in UDI domain scores were compared. The UDI scores range from 0 to 100, with lower scores indicating less bother caused by urogenital symptoms. To facilitate interpretation of the clinical relevance of changes in UDI, effect sizes were calculated.

Lastly, patients were asked if they underwent any urogenital re‐operation.

### 2.3 Statistical Analysis

We expected a limited response rate inherent to long‐term outcome studies. Therefore, we decided to perform two separate analyses; one for women who responded to the questionnaire, referred to as ‘responders only’, and one for all women who entered the study, referred to as ‘all women’. For those who did not respond, referred to as ‘non‐responders’, outcomes at 12 months were used for the last observation carried forward principle. Treatment effects on binary variables, including PGI‐I [improvement], PGI‐S [no symptoms] and subjective cure [cure], are presented as absolute change in percentage points between groups with 95% confidence interval (CI). Statistical significance was assessed using Fisher's exact test. Furthermore, for continuous data, change in UDI score over time within each treatment strategy was analysed using a paired‐samples Student's *t*‐test. An independent Student's *t*‐test was used to compare mean changes in UDI scores between the treatment strategies. To aid the interpretation of the changes, effect sizes were calculated with the use of Cohen's *d* test. An effect size of 0.3 or less was considered small, more than 0.3–0.8 moderate, and more than 0.8 large [18]. All statistical analyses were performed with the use of IBM SPSS Statistics [19].

## 3 Results

### 3.1 Study Population

**FIGURE 1:**
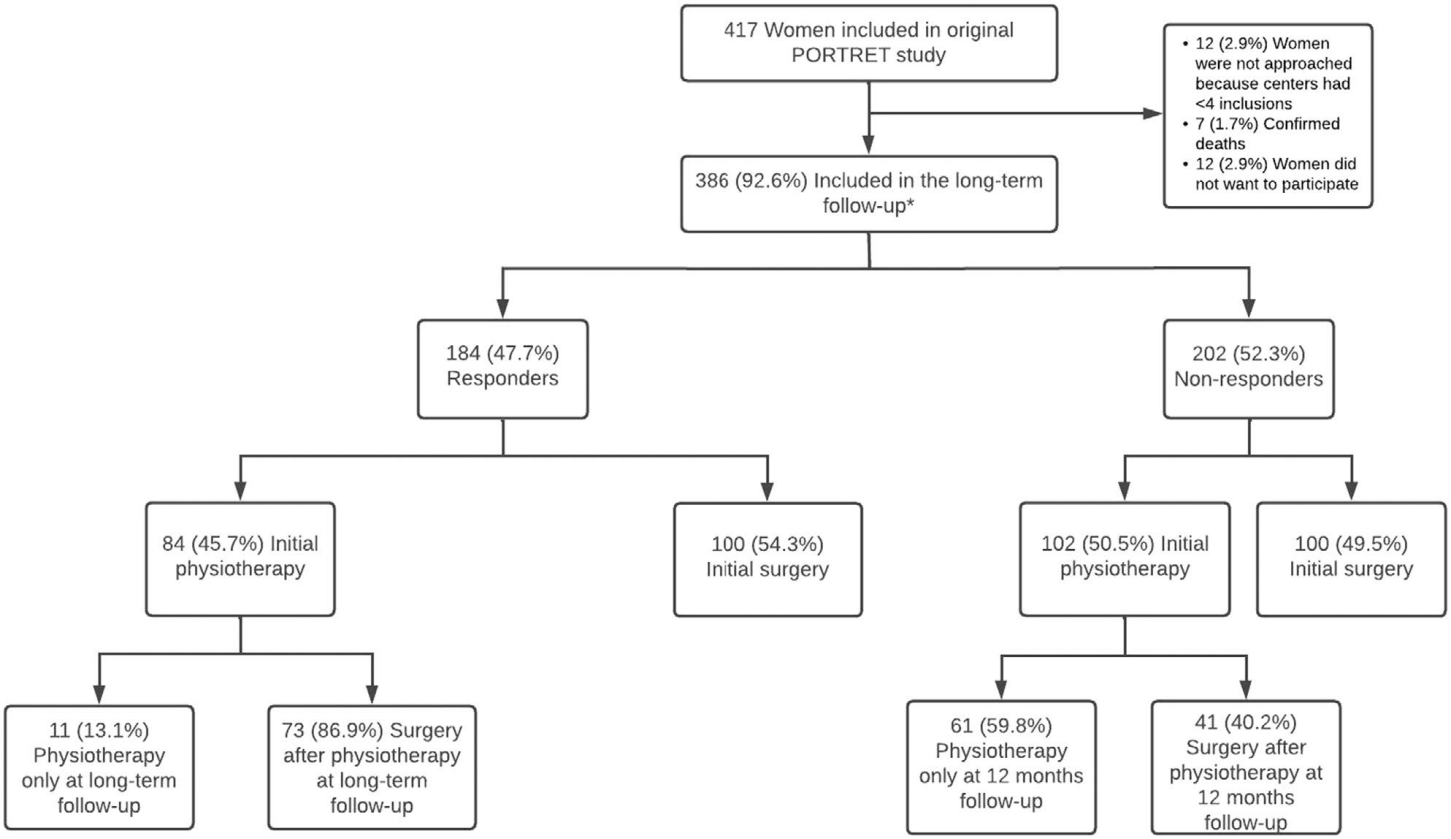
Long term follow‐up. *186 initial physiotherapy and 200 initial surgery.

Data in the PORTRET study was collected from March 2008 through May 2010 [4]. In our long‐term follow‐up, data was collected from September 2020 through March 2023. The mean follow‐up time in our study was 11.9 years, with a range from 10.5 to 14.8 years. Flowchart of the long‐term follow‐up is shown in Figure 1.

In the PORTRET study, 417 women were originally analysed [4]. Firstly, 12 women were excluded because they were not approached based on < 4 inclusions in the original participating hospital. Of the 405 women contacted, 7 were reported to have died, and 12 women actively responded that they did not want to participate, leaving 386 women to be included in our study (200 initial surgery and 186 initial physiotherapy). Of these 386 women, 184 filled in the questionnaire, resulting in a 47.7% response rate. Of these 184 responders, 84 women were originally randomised in the physiotherapy group and 100 women in the surgery group. In the physiotherapy group, a total of 73 women (86.9%) reported to have crossed over to surgery and 11 women received physiotherapy only. In the initial surgery group, 6 (6%) had additional physiotherapy due to urinary incontinence problems; four had SUI symptoms and two had urge urinary incontinence (UUI) symptoms. For the non‐responders, the flow chart shows the distribution of their treatment modalities at 12 months follow‐up in the original trial.

### 3.2 Intention to Treat Analysis

Baseline characteristics were similar in both original treatment groups and have been published previously [4]. Detailed baseline characteristics, stratified by responder status and allocated treatment, can be found in the Supporting Information (Table S1). Primary and secondary outcomes according to randomisation are presented in Table 1 for both the responders only as well as for all women. In the analysis of the responders only, no statistically significant differences were found in any outcomes between women who underwent initial surgery or initial physiotherapy. In the analysis of all women, subjective improvement (absolute difference 20.8%; 95% CI 12.1–29.6), severity of incontinence (absolute difference 13.0%; 95% CI 3.4–22.5), and subjective cure (absolute difference 23.8%; 95% CI 13.9–33.7) statistically significantly improved in favour of initial surgery compared to initial physiotherapy. Women in the initial surgery group improved statistically significantly more compared to the women in the initial physiotherapy group on the urinary incontinence domain of the UDI, with a moderate effect size (0.38; 95% CI 0.12–0.59). Other domains of the UDI were not statistically significant different between the groups.

**TABLE 1:** Intention to treat with primary and secondary outcomes in the surgery and physiotherapy group for responders only and all women at long‐term follow‐up.

Intention to treat analysis								
Outcome	Responders only (*n* = 184)				All women (*n* = 386)			
	Initial surgery (*n* = 100)	Initial physiotherapy (*n* = 84)	Absolute difference (95% CI)	*p*	Initial surgery (*n* = 200)	Initial physiotherapy (*n* = 186)	Absolute difference (95% CI)	*p*
PGI‐I: improvement—no./total no. (%)	87/100 (87.0)	66/83 (79.5)	− 7.5 (−18.5 to 3.6)	0.229	163/188 (86.7)	108/164 (65.9)	20.8 (12.1 to 29.6)	< 0.001
PGI‐S: no symptoms—no./ total no. (%)	70/100 (70.0)	62/84 (73.8)	3.8 (−9.4 to 17)	0.624	144/188 (76.6)	105/165 (63.6)	13.0 (3.4 to 22.5)	0.010
Subjective cure—no./ total no. (%)	66/91 (72.5)	51/75 (68.0)	4.5 (−9.7 to 18.7)	0.320	142/180 (78.9)	86/156 (55.1)	23.8 (13.9 to 33.7)	< 0.001
Change in UDI domain score^a^								
Urinary incontinence	−31.4 (22.2)	−29.3 (22.8)	2.1 (−4.5 to 8,7)	0.531	−35.1 (20.2)	−26.6 (24.8)	8.5 (3.7 to 13.3)	< 0.001
Overactive bladder	−4.4 (24.1)	−2.6 (21.1)	1.8 (−4.9 to 8.5)	0.597	−11.5 (21.0)	−9.3 (19.4)	2.2 (−2.1 to 6.5)	0.313
Obstructive micturition	0.7 (25.2)	2.0 (22.3)	1.3 (−5.7 to 8.4)	0.708	−4.8 (21.3)	−1.7 (20.1)	3.0 (−1.3 to 7.4)	0.174
Discomfort or pain	−3.3 (15.3)	0.2 (13.1)	3.4 (−7.8 to 7.6)	0.110	−5.8 (13.0)	−4.1 (11.9)	1.7 (−1.0 to 4.3)	0.217
Genital prolapse	0.3 (18.7)	0.8 (19.5)	0.5 (−5.1 to 6.1)	0.870	−1.2 (11.8)	−1.2 (12.4)	0.1 (−2.5 to 2.6)	0.960

^a^ Data regarding change in UDI domain score for all women were available for 187 women in the initial surgery group and 162 women in the initial physiotherapy group. Values are means (SD).

### 3.3 Post Hoc Analysis

Primary and secondary outcomes according to the three treatment modalities are presented in Table 2 for both the responders only as well as for all women. The long‐term follow‐up shows that subjective improvement was statistically significant more often reported by women who underwent surgery as compared to physiotherapy only; both for women who underwent initial surgery (absolute difference 50.6% (95% CI 28.2–73) for responders only and 62.1% (95% CI 49.7–74.6) for all women), as well as for women who crossed over from physiotherapy to surgery (absolute difference 49.7% (95% CI 25.8–73.7) for responders only and 63.3% (95% CI 50.2–76.3) for all women). In addition, severity of incontinence statistically significantly improved among women who underwent surgery compared to physiotherapy only; both for women who underwent initial surgery (absolute difference 33.6% (95% CI 4.4–62.9) for responders only and 50.3% (95% CI 37.5–63.1) for all women), as well as for women who crossed over from physiotherapy to surgery (absolute difference 43.1% (95% CI 16.1–70.1) for responders only and 57.0% (95% CI 43.3–70.7) for all women). Furthermore, the subjective cure rate was statistically significantly higher among women who underwent surgery compared to physiotherapy only; both for women who underwent initial surgery (absolute difference 52.5% (95% CI 23.0–82.0) for responders only and 66.4% (95% CI 55.7–77.1) for all women), as well as for women who crossed over from physiotherapy to surgery (absolute difference 55.4% (95% CI 26.1–84.7) for responders only and 66.5% (95% CI 54.5–78.5) for all women).

**TABLE 2:** Post hoc analysis of primary and secondary outcomes for responders only and all women at long‐term follow‐up.

Post hoc analysis												
Outcome	Responders only (*n* = 184)						All women (*n* = 386)					
	A: Initial Surgery (*n* = 100)	B: Physiotherapy only (*n* = 11)	C: Surgery after physiotherapy (*n* = 73)	Absolute difference (95% CI)			A: Initial Surgery (*n* = 200)	B: Physiotherapy only (*n* = 72)	C: Surgery after physiotherapy (*n* = 114)	Absolute difference (95% CI)		
				A versus B	A versus C	B versus C				A versus B	A versus C	B versus C
PGI‐I: improvement—no./total no. (%)	87/100 (87.0)	4/11 (36.4)	62/72 (86.1)	50.6 (28.2 to 73.1)	−0.9 (−11.3 to 9.6)	49.7 (25.8 to 73.7)	163/188 (86.7)	14/57 (24.6)	94/107 (87.9)	62.1 (49.7 to 74.6)	−1.2 (−9.2 to 6.9)	63.3 (50.2 to 76.3)
PGI‐S: no symptoms—no./total no. (%)	70/100 (70.0)	4/11 (36.4)	58/73 (79.5)	33.6 (4.4 to 62.9)	9.5 (−3.9 to 22.8)	43.1 (16.1 to 70.1)	144/188 (76.6)	15/57 (26.3)	90/108 (83.3)	50.3 (37.5 to 63.1)	−6.7 (−16.1 to 2.6)	57.0 (43.3 to 70.7)
Subjective cure—no./total no. (%)	66/91 (72.5)	2/10 (20.0)	49/65 (75.4)	52.5 (23.0 to 82.0)	−2.8 (−17.1 to 11.3)	55.4 (26.1 to 84.7)	142/180 (78.9)	7/56 (12.5)	79/100 (79.0)	66.4 (55.7 to 77.1)	−0.1 (−10.2 to 9.9)	66.5 (54.5 to 78.5)
Change in UDI domain score^a^												
Urinary incontinence	−31.4 (22.2)	−11.3 (15.6)	−32.1 (22.6)	20.2 (6.5 to 33.8)	0.7 (−7.5 to 6.2)	−20.8 (−34.9 to −6.8)	−35.1 (20.2)	−7.7 (19.6)	−36.4 (21.2)	27.4 (21.4 to 33.4)	−1.3 (−6.2 to 3.6)	−28.7 (−35.5 to −22.0)
Overactive bladder	−4.4 (24.1)	1.5 (27.2)	−3.2 (20.1)	5.9 (−9.5 to 21.3)	1.2 (−5.8 to 8.9)	−4.7 (−18.4 to 8.9)	−11.5 (21.0)	−6.6 (17.7)	−10.7 (20.1)	4.9 (−1.2 to 10.9)	0.8 (−4.1 to 5.7)	−4.0 (−10.4 to 2.3)
Obstructive micturition	0.7 (25.2)	3.0 (19.5)	1.9 (22.8)	2.4 (−13.2 to 17.9)	1.2 (−6.2 to 8.6) 4	−1.2 (−15.6 to 13.3)	−4.8 (21.3)	0.9 (21.8)	−3.1 (19.1)	5.7 (−0.8 to 12.1)	1.7 (−3.2 to 6.5)	−4.0 (−10.6 to 2.5)
Discomfort or pain	−3.3 (15.3)	2.4 (8.7)	−0.2 (13.7)	5.7 (−3.6 to 15.0)	3.1 (−1.4 to 7.5)	−2.6 (−11.1 to 5.9)	−5.8 (13.0)	−3.7 (10.2)	−4.3 (12.6)	2.1 (−1.7 to 5.9)	1.4 (−1.6 to 4.5)	−0.7 (−4.6 to 3.2)
Genital prolapse	0.3 (18.8)	4.5 (10.8)	0.2 (20.5)	4.2 (−7.2 to 15.6)	0.1 (−6.1 to 5.9)	−4.3 (−16.9 to 8.3)	−1.2 (11.8)	−0.8 (7.6)	−1.4 (14.2)	0.5 (−2.8 to 3.8)	−0.2 (−3.2 to 2.9)	−0.6 (−4.7 to 3.4)

^a^ Data regarding change in UDI domain score for all women were available for 187 women in the surgery group, 55 women in the physiotherapy only group and 107 women in the surgery after physiotherapy group. Values are means (SD).

Regarding urogenital symptoms on the five domains of the UDI, the only statistically significant differences were found in the urinary incontinence domain. Women who underwent surgery, initially or after physiotherapy, improved significantly more on this UDI domain compared to women who underwent physiotherapy only. The effect sizes were large both for responders only (physiotherapy only compared to initial surgery 0.93 (95% CI 0.29–1.56) and physiotherapy only compared to surgery after physiotherapy 0.95 (95% CI 0.30–1.60)) as well as for all women (physiotherapy only compared to initial surgery 1.36 (95% CI 1.04–1.69) and physiotherapy only compared to surgery after physiotherapy 1.39 (95% CI 1.03–1.74)). No statistically significant differences were found in the other UDI domains between the treatment modalities.

### 3.4 Re‐Operation

Long‐term urogenital re‐operations were reported by 8 of 173 responders that received mid‐urethral sling surgery (4.6%): 5 were due to a complication from surgery, 2 due to recurrence of urinary incontinence and 1 was due to recurrence of urinary incontinence combined with a complication of surgery.

## 4 Discussion

### 4.1 Main Findings

In this 12‐year long‐term outcome study of women previously included in the PORTRET study, we found a high cross‐over rate (86.9%) from initial physiotherapy to surgery. In the intention to treat analysis for those who responded, we could not demonstrate statistically significant differences between groups, but this was obviously biased by the high cross‐over rate to surgery. In the post hoc analysis we compared three treatment modalities; initial surgery, physiotherapy after surgery and physiotherapy only. We demonstrated that women with moderate to severe urinary stress incontinence experienced statistically significantly better subjective outcomes after surgery, either initially or after physiotherapy, compared to those who received physiotherapy only. In addition, treatment with physiotherapy before surgery did not result in additional benefits compared to surgery alone. The high effect sizes of the UDI urinary incontinence domain between women who underwent physiotherapy only and (initial) surgery indicate a strong clinical relevance of our findings. Re‐operation was reported by 8/173 (4.6%) of women.

### 4.2 Strengths and Limitations

This study presents long‐term follow‐up data of a Dutch nationwide, multi‐centre, randomised controlled trial, where cross‐over was allowed. In addition, validated outcome measures were used, such as the PGI‐I and PGI‐S [11, 12, 13]. We focused solely on patient‐reported outcome measures, as these are the most reliable indicators of symptom‐related distress, often underestimated by health care providers [20, 21]. Women who crossed over to surgery in the long‐term were not actively approached for further treatment, ensuring that their decision to undergo surgery in the long‐term was made independently and without external influence. A limitation of our study was the absence of monitoring long‐term adherence of responders to pelvic floor muscle therapy. It could be argued that a lack of adherence compromises the validity of the comparison, as patients who do not adhere to physiotherapy may not fully realise the potential benefits of the exercises. However, non‐adherence may also indicate the limited effect of the physiotherapy itself, as patients may discontinue treatment if they perceive minimal benefit. Moreover, by assessing adherence retrospectively, recall bias would have been a limitation in interpreting our results. By focusing on treatment strategy, rather than solely on the treatment itself, our study reflects the outcomes that are likely to occur in routine clinical practice, where adherence to physiotherapy without any form of extra motivation may vary. Overall, we believe our approach provides valuable insights into the daily practice of physiotherapy as an initial treatment strategy for moderate to severe SUI. Furthermore, another limitation of our study was the limited sample size, due to the 47.7% response rate to the questionnaire. In order to account for this we decided to use the last observation carried forward principle for the non‐responders and impute this data into our database by the all women analysis. Nevertheless, this approach may underestimate the cross‐over rate or overestimate outcomes. This has to be recognised as a limitation, although it probably shows the most positive scenario for physiotherapy.

### 4.3 Interpretation

Long‐term patient improvement rates following initial surgery were 87.0% among responders only and 86.7% among all women, which is consistent with existing literature reporting 87%–89% improvement rates after 17–20 years follow‐up of midurethral sling surgery [5, 6, 7]. Patient improvement rates following initial physiotherapy were 79.5% among responders only and 65.9% among all women. However, this effect is most likely largely attributed to the effect of surgery, due to the high cross‐over rate to surgery. Consequently, the subjective improvement rates in the physiotherapy only group in the post hoc analysis, 36.4% (responders only) and 24.6% (all women), likely provide a more accurate assessment of the effectiveness of physiotherapy. Bø et al. reported a 41%–85% improvement in SUI complaints with physiotherapy over the long‐term, with a wide variety of long‐term adherence [9]. Our post hoc analyses showed lower improvement rates for the physiotherapy only group, possibly because treatment success depends on patient adherence, which is challenging to maintain and monitor especially in the long‐term [8, 9].

Moreover, in the post hoc analysis subjective improvement in the surgery after physiotherapy group was similar compared to the initial surgery group. These results are in line with research by Sung et al., who found no clinically significant difference in urinary symptoms at 12 months when combining pelvic floor muscle therapy with midurethral sling surgery compared to sling surgery alone [22]. One might argue that promoting a treatment strategy to women that starts with physiotherapy is justified by its perceived safety, given the lack of negative side effects. However, since the beneficial effects are not proven, counselling women with accurate outcome figures for all treatment modalities could prevent a delay in receiving the most effective treatment.

Furthermore, it is noticeable that in our study there was no statistically significant increase in overactive bladder symptoms after surgery. Long‐term literature reports de novo overactive bladder complaints in 9%–42% of women undergoing surgery [6, 7, 23]. This discrepancy in results may be due to different definitions, or single‐item versus domains scores used in these questionnaires for overactive bladder symptoms.

Regarding cross‐over rates, 49.0% of women crossed over after 12 months follow‐up [4]. In this long‐term follow‐up, 86.9% of responders and at least 61.3% of all women crossed over to surgery, assuming all 61 non‐responders who received physiotherapy only at 12 months follow‐up did not cross‐over to surgery in the long term. This indicates that even in the long‐term, women tend to cross‐over to surgery on their own initiative. Literature reports long‐term surgery rates after physiotherapy ranging from 4.9% to 58% [9]. Our cross‐over rate is higher, possibly due to our selection bias. We only included women with moderate to severe symptoms and excluded those with mild symptoms, and women with a preference for surgery might have been more likely to participate in the PORTRET study. Participating allowed them the chance to go to surgery directly instead of having to follow the regular protocol of physiotherapy first [4]. However, this possible selection bias is expected to be less present in the long‐term, as women who preferred surgery probably would have crossed over earlier.

Lastly, in this long‐term follow‐up urogenital re‐operation was reported by 8/173 (4.6%) of women, primarily due to complications after surgery. The frequency of re‐operations was comparable with the 3%–5% described in literature [24, 25].

## 5 Conclusion

At the 12‐year follow‐up of the PORTRET study, there was a very high cross‐over rate from physiotherapy to surgery, taking into account a considerable proportion of non‐responders. Women experienced statistically significantly better subjective outcomes for moderate to severe SUI after midurethral sling surgery, either initially or after physiotherapy, compared to women who underwent pelvic floor muscle physiotherapy only. Pelvic floor muscle therapy prior to surgery did not augment outcomes compared to initial surgery and no differences in overactive bladder or urogenital symptoms were found between the treatment modalities. Consequently, patients should be informed on the persistent higher satisfaction associated with surgery, the likelihood of cross‐over to surgery following initial physiotherapy, and the low probability of needing an urogenital re‐operation after mid‐urethral sling surgery in the long‐term. In practice, we consider it important to inform women with moderate to severe SUI of these long‐term results and offer them the option having midurethral sling surgery, even without prior physiotherapy.

## Author Contributions

Carl. H van der Vaart together with Deodata Tijsseling and Julien Labrie conceptualised the trial. Deodata Tijsseling made the study protocol and together with Hélène F.C. van Oorschot led application for the ethics committee. Julien Labrie advised on methodology, as he was the chief investigator of the original PORTRET study. Deodata Tijsseling and Hélène F. C. van Oorschot were responsible for the acquisition of data by working together with all centres involved. Hélène F.C. van Oorschot was responsible for all the data analysis and interpretation of data, with valuable contributions and support from Deodata Tijsseling and Julien Labrie. Consequently, Hélène F.C. van Oorschot drafted the article, which was reviewed and edited by all authors. All authors approved for submission and are accountable for all aspects of the work. Hélène F. C. van Oorschot is the guarantor.

## Ethics Statement

The Medische Ethische Toetsingscommisie (METC) (WAG/mb/20/013631) from the Universitair Medisch Centrum Utrecht (UMCU) approved to conduct a non‐WMO research on the 8th of April 2020 (protocol number 20‐201/C); stating this to be a low‐risk study with consent from participants, where anonymised patient data was used.

## Conflicts of Interest

All authors of this article have no conflicts of interest to declare. The study group has no competing financial, professional, or personal interests that might have influenced the study design or conduct.

## Supporting information


Table S1.

## Data Availability

The data that support the findings of this study are available on request from the corresponding author. The data are not publicly available due to privacy or ethical restrictions.
