# Supplementary material for: Twelve‐Year Follow‐Up of a Randomised Controlled Trial Comparing the Effectiveness of Pelvic Floor Muscle Training Versus Mid‐Urethral Sling Surgery for Female Moderate to Severe Urinary Incontinence
Source: BJOG. 2025 Feb 11;132(6):826–33. doi: 10.1111/1471-0528.18092 (PMC11969909; doi:10.1111/1471-0528.18092)
Supplement: Supplementary file 1 — Table S1. [file BJO-132-826-s001.docx]

Table S1: Baseline characteristics of the study population stratified by responder status and allocated treatment.

|  | **Responders (n=184)** | | **Non-responders (n=202)** | |
| --- | --- | --- | --- | --- |
|  | Initial surgery (n=100) | Initial physiotherapy (n=84) | Initial surgery (n=100) | Initial physiotherapy (n=102) |
| Age (years) – mean (SD) | 47.9 (9.0) | 49.8 (7.1) | 51.6 (9.5) | 49.9 (9.1) |
| College or university degree – no./total no. (%) | 32/99 (32.3) | 22/84 (26.2) | 23/98 (23.5) | 24/99 (24.4) |
| **Parity *** |  | | | |
| Median | 2 | 2 | 2 | 2 |
| Range | 0-4 | 1-5 | 1-4 | 0-7 |
| Current smoker – no./ total no. (%) | 15/97 (15.5) | 12/79 (15.2) | 17/96 (17.7) | 20/96 (20.8) |
| Body mass index - mean (SD)** | 26.9 $\boldsymbol{\pm}$ 5.7 | 26.7 $\boldsymbol{\pm}$ 4.4 | 26.0 (4.2) | 27.0 (5.5) |
| Postmenopausal – no./total no. (5) | 31/99 (31.3) | 28/83 (33.7) | 42/97 (43.3) | 33/97 (34.0) |
| **No. voidings in 24h period ***** |  | | | |
| Median | 8 | 8 | 8 | 8 |
| Range | 3-19 | 4-17 | 4-22 | 3-14 |
| Physiotherapy >6 mo before study – no./ total no. (%) | 19/99 (19.2) | 19/81 (23.5) | 15/95 (15.8) | 17/95 (17.9) |
| PGI-S: not severe – no./ total no. (%) | 5/100 (5.0) | 8/83 (9.6) | 4/99 (0.4) | 4/98 (0.4) |
| **UDI domain score - mean (SD) ****** |  | | | |
| Urinary incontinence | 44.4 (20.8) | 42.6 (18.1) | 40.7 (18.0) | 39.5 (19.4) |
| Overactive bladder | 21.7 (21.2) | 16.8 (19.6) | 22.9 (22.0) | 18.9 (18.5) |
| Obstructive micturition | 16.3 (22.5) | 11.2 (17.5) | 13.1 (18.6) | 9.8 (17.4) |
| Discomfort or pain | 12.9 (16.0) | 7.6 (11,3) | 8.8 (11.8) | 11.3 (14.4) |
| Genital prolapse | 4.3 (2.4) | 14.2 (12.5) | 3.9 (9.2) | 2.7 (8.1) |

Data were available for:

*Responders: 98 women in the initial surgery group and 82 women in the initial physiotherapy group. Non-responders; 96 women in the initial surgery group and 99 women in the initial physiotherapy group.** Responders: 81 women in the initial physiotherapy group. Non-responders: 98 women in the initial surgery group and 97 in the initial physiotherapy group. *** Responders: 96 women in the initial surgery group and 75 women in the initial physiotherapy group. Non-responders: 94 women in the initial surgery group and 89 women in initial physiotherapy group.****Responders: 99 women in the initial surgery group and 83 women in the initial physiotherapy group. Non-responders: 99 women in the initial surgery group and 100 women in the initial physiotherapy group.
